# Prevalence, perceptions and factors associated with non-adherence to hepatotoxicity monitoring among people living with HIV on tuberculosis preventive treatment at Mulago ISS clinic

**DOI:** 10.1371/journal.pone.0345662

**Published:** 2026-03-30

**Authors:** Kevin Naturinda, Irene Andia Biraro, Ezekiel Mupere, Anthony Muyunga, Racheal Namutale Nalule, Benard Owori, Linda Dorothy Akot, Jovan Mugerwa, Arthur Shem Kasambula, Joseph Rubeihayo, Joan Kalyango

**Affiliations:** 1 Clinical Epidemiology Unit, School of Medicine, College of Health Sciences, Makerere University, Kampala, Uganda; 2 Department of Internal Medicine, School of Medicine, College of Health Sciences, Makerere University, Kampala, Uganda; 3 Department of Paediatrics and Child Health, School of Medicine, College of Health Sciences, Makerere University, Kampala, Uganda; 4 Department of Immunology and Molecular Biology, School of Biomedical Sciences, College of Health Sciences, Makerere University, Kampala, Uganda; 5 Department of Pharmacy, School of Health Sciences, College of Health Sciences, Makerere University, Kampala, Uganda; UN Women, INDIA

## Abstract

**Introduction:**

Hepatotoxicity monitoring is recommended for people living with HIV (PLHIV) enrolled on tuberculosis preventive treatment (TPT). Despite a 7.3% hepatotoxicity incidence reported in a multi-country study, implementation and adherence to hepatotoxicity monitoring remains low. We aimed at determining the prevalence, patients’ and health workers’ perceptions, and factors associated with non-adherence to hepatotoxicity monitoring among adult PLHIV receiving TPT at Mulago ISS clinic.

**Methods:**

A sequential explanatory mixed methods study was carried among health workers and adult PLHIV enrolled on TPT between 2022 and 2023 at Mulago ISS clinic. The quantitative and qualitative components utilized a cross-sectional and an exploratory descriptive design respectively. A data abstraction tool was used to obtain quantitative data from files and electronic medical records of a systematic random sample of 390 patients. The qualitative study utilized interview guides to conduct in-depth audio recorded interviews for five patients and five health workers. Descriptive statistics and modified Poisson regression analyses were performed for the quantitative data. Deductive thematic analysis based on the health belief model was utilized for the qualitative data.

**Results:**

The prevalence of non-adherence to hepatotoxicity monitoring was 87.4% (95%CI: 83.7–90.4). The prevalence of non-adherence to hepatotoxicity monitoring was 85% lower in PLHIV that received primary TPT as compared to those on secondary TPT (aPR-0.15, 95% CI: 0.04–0.55). The qualitative findings revealed varied perceptions about non-adherence to hepatotoxicity monitoring that included awareness of associated risks, perceived benefits of monitoring, and confidence in taking action, while also identifying perceived barriers such as financial, knowledge and communication challenges.

**Conclusions:**

The prevalence of non-adherence to hepatotoxicity monitoring was high within this pharmacovigilance sentinel site, given its clinical importance in HIV care and clear recommendations in the national HIV treatment guidelines. Health authorities should implement policies promoting hepatotoxicity monitoring in pharmacovigilance sentinel sites, especially for patients receiving secondary TPT, and allocate resources to support this initiative.

## Introduction

Persons living with HIV (PLHIV) are at risk of developing tuberculosis (TB) with 0.66 million TB incident cases attributed to HIV infection, and 161,000 TB related deaths among PLHIV in 2023 globally [1]. TB preventive treatment (TPT) use is recommended by the World Health Organization (WHO) to reduce the burden of TB among HIV patients [2]. However, this intervention carries a risk of hepatotoxicity, making its monitoring crucial for proper management of patients [3]. Timely hepatotoxicity monitoring is crucial for early detection of hepatotoxicity, thus preventing undetected liver damage, reducing on avoidable hospitalization and poor patient treatment outcomes like treatment discontinuation and mortality [4,5]. Previous studies have indicated that the development of hepatotoxicity, which is shown by elevated liver enzymes, is significantly associated with TPT use in PLHIV [6]. A multi-country open-label randomized control clinical trial reported the incidence of hepatotoxicity among PLHIV on TPT to be 7.3% [6]. Whereas, a study conducted in 3 tertiary hospitals in Uganda reported the prevalence of isoniazid-related hepatotoxicity among patients on TPT at 7% [7]. The high prevalence may be partly attributed to inadequate baseline liver assessment and limited follow-up hepatotoxicity monitoring, which can delay detection of mild hepatotoxicity that later progresses to more severe forms. To maintain low levels of hepatotoxicity, a guideline had been drafted where patients on TPT receiving care from pharmacovigilance (PV) sentinel sites are required to be monitored for hepatotoxicity by carrying out liver function tests (LFTs) at baseline, 3 months after initiation of TPT, and when requested to do the test by the clinician after patient assessment on any visit [5]. Monitoring for hepatotoxicity can be done through laboratory testing, history taking and physical examination for signs suggestive of hepatic injury. Despite monitoring guidelines, there are still gaps in both the implementation and adherence to the recommended hepatotoxicity monitoring. Laboratory based monitoring is limited by resources thus, clinicians may prefer other monitoring approaches, which may not be sensitive enough to detect asymptomatic hepatotoxicity. Mulago ISS clinic being a PV sentinel site, provides LFTs at no cost to patients through the clinic; however, during reagent stock-outs, patients may obtain the test privately at an average cost of approximately forty-five thousand Ugandan shillings. Additionally, the patient and clinician perceptions seem to contribute to low adherence; for example, clinicians tend to underestimate the risk of hepatotoxicity and instead prioritize other aspects of patient care over monitoring for hepatotoxicity. Studies have been done to determine the burden of hepatotoxicity, but very few are investigating the adherence to the hepatotoxicity monitoring guidelines in PV sentinel sites. Therefore, this study aimed at determining the prevalence, patients’ and health workers’ perceptions, and factors associated with non-adherence to hepatotoxicity monitoring among adult PLHIV receiving TPT at Mulago ISS clinic, Kampala, Uganda.

## Methods

### Study design

A sequential explanatory mixed methods study was conducted between 17^th^ April 2024 and 30^th^ May 2024. In the first phase, a quantitative cross-sectional study was carried out to determine the prevalence and factors associated with non-adherence to hepatotoxicity monitoring. In the second phase, a qualitative exploratory descriptive study was conducted, guided by the quantitative findings, to provide in-depth explanations and contextual understanding of the patterns observed.

### Study site

The study was conducted at Mulago ISS clinic. The facility is supported and operated by the Makerere University Joint AIDS Program (MJAP), which is a private not-for-profit (PNFP) non-governmental organization (NGO) that offers free comprehensive HIV/AIDS services. Mulago ISS clinic is located within Mulago National referral and Teaching Hospital complex along Upper Mulago Hill Road within Kawempe south division in Kampala district, Uganda’s capital city. It’s a hub for Implementation Science research on HIV health service delivery, HIV and TB diagnosis, care/treatment. It is a large single HIV clinic and a Centre of excellence, providing comprehensive HIV services to about 17,000 PLHIV and about 6,000 PLHIV enrolled on TPT in 2022 and 2023, of whom 2,563 aged at least 18 years were enrolled on either isoniazid (INH) or isoniazid and rifapentine once weekly for 12 weeks (3HP).

### Study population

The quantitative study was conducted among PLHIV aged at least 18 years who had initiated and completed their TPT regimen between January 2022 and December 2023 at Mulago ISS clinic. We excluded participants whose records could not be traced. The qualitative study was conducted among PLHIV aged at least 18 years that had enrolled on TPT in 2022 and 2023, and health workers that were engaged in management of PLHIV enrolled on TPT at Mulago ISS clinic. Patients that were critically ill and those that could not communicate in either Luganda or English were excluded from the qualitative study.

### Sample size and sampling procedure for the study participants

The sample size for the quantitative study was estimated basing on the formula for a single proportion by Leslie Kish [8,9]. The parameters assumed were 95% level of confidence, estimated prevalence of non-adherence to hepatotoxicity monitoring of 0.558 as reported in study conducted in Taiwan [10], and 5% tolerable sampling error that yielded a sample size of 379 patient records. In addition, for the aspect of factors associated with non-adherence to hepatotoxicity monitoring, the sample size was computed using the formula for comparing of two proportions that yielded a sample size 222 patient records [11]. The parameters included 5% level of significance, 80% power of the study, 0.07 as the proportion of patients with a hepatotoxicity adverse reaction while on TPT, 0.93 as the proportion of patients with non-hepatotoxicity adverse drug reaction while on TPT, 0.96 as the proportion of patients with hepatotoxicity that have not been monitored for liver function and 0.672 as the assumed proportion of patients with non-hepatotoxicity adverse drug reaction that have not been monitored for liver function aimed at achieving a 30% clinically significant difference [7]. The sample size (379) obtained from the computation using the single proportion formula was considered for this study. This is because it was the highest and also adequate to determine the factors associated with non-adherence to hepatotoxicity monitoring. However, in anticipation of missing data, a total of 400 patient records were considered for the study. Systematic random sampling was utilized to select the participants. A total of 2,563 eligible patient records were retrieved from electronic medical records (EMR). A sampling interval of seven (7) was obtained by dividing 2,563 by the sample size (400). The first record in the sample was randomly selected from the first seven records. Thereafter, every 7^th^ record was included in the sample. A total of 390 records (10 records short of the targeted 400 records) were traced and included in the analysis. For the qualitative study, data collection was done until saturation which was achieved at 10 interviews. Purposive sampling was deployed while utilizing maximum variation strategy to select five health workers (two clinical officers, two doctors and one laboratory personnel) with varying work experience, and five patients with varying gender, age, level of education and occupation at the time they received TPT.

### Data collection

In the quantitative component of the study, trained research assistants used pre-designed and tested data abstraction tools to extract anonymized demographic, behavioral and clinical data as well as data on hepatotoxicity monitoring. They extracted the data from the EMR and patient files between 17^th^ April 2024 and 17^th^ May 2024. The behavioral and demographic data included age, sex, level of education, marital status, employment status and area of residence. The clinical data included antiretroviral therapy (ART) regimen, baseline HIV viral load and CD4 count, TPT regimen, Type of TPT (it was defined as Primary TPT if the patient was enrolled for TPT for the first time since ART initiation, and Secondary TPT if the patient was enrolled for TPT for the second or subsequent time since ART initiation), Having Hepatitis B result, WHO HIV/AIDS clinical stage [12], history of adverse drug reactions and presence of co-morbidities (liver disease, hypertension or diabetes). The dependent variable was “non-adherence to hepatotoxicity monitoring” which was defined as not having performed LFTs prior to TPT initiation, 3 months after initiation of TPT and any other time while the patient was on TPT. However, since the recommended adherence to hepatotoxicity monitoring for PV sentinel-sites was not observed in the study population, adherence was operationalized as receipt of at least one documented LFT during the course of TPT. Participant who had not performed LFTs at any of the mentioned occasions were categorized as non-adherent and coded as “1”, while those that had performed LFTs at least once, were categorized as adherent and coded as “0” for analysis.

For the qualitative component, the principal investigator (PI) and the research assistants used pre-tested interview guides to conduct audio-recorded in-depth interviews. We collected data on perceptions about non-adherence to hepatotoxicity monitoring to further explain its prevalence. A total of 10 audio-recorded interviews were conducted from 20^th^ to 28^th^ May 2024. Eight (03 patients and 5 health workers) were conducted in English and two (all for patients) in Luganda, the most common local language spoken in the study area. The interview sessions lasted for 15–20 minutes and were all conducted from a quiet doctor’s room either in the morning before routine work or in the evening after work.

### Data management and statistical analysis

Quantitative data was entered into a predesigned electronic data capture tool in Epi-data version 3.1. Data was then imported into STATA version 14 for cleaning and analysis. Descriptive statistics were expressed as either mean (+/- standard deviation (SD)) or median (with 1^st^ quartile (Q1) and 3^rd^ quartile (Q3)) for numerical variables, while categorical variables were expressed as percentages. The prevalence of non-adherence to hepatotoxicity monitoring was determined as a percentage of participants who were classified as non-adherent to hepatotoxicity monitoring, presented with its 95% confidence interval. Modified poisson regression model with robust standard errors, from the generalized linear model (GLM) window, was used to determine the factors associated with non-adherence to hepatotoxicity monitoring. The prevalence ratio was the measure of association estimated. All variables with a p-value <0.2 at bivariate analysis were considered for multivariate analysis. Variables were assessed for interaction using the likelihood ratio test (chunk test) that compared full and reduced models. Confounding was assessed for variables with no interaction and not significant in the model by comparing the prevalence ratios from the crude and adjusted models. Variables that caused at least 10% change in the prevalence ratio were retained as confounders. Statistical significance of the final model was set at 0.05 level of significance.

For the qualitative data, audio recorded interviews were transcribed verbatim and Luganda transcripts were translated into English and transcribed at the same time. Data was analyzed using Open Code version 4.02 using deductive thematic analysis, guided by the health belief model (HBM). The PI thoroughly read the transcripts to get familiarized with the data before he could generate codes from the relevant data sections. An independent researcher reviewed and refined the codes for accuracy and consistence. Triangulation of the qualitative and quantitative findings was done at the discussion stage as the qualitative findings were used to explain the quantitative findings.

### Ethical considerations

We obtained ethical clearance from School of Medicine Research Ethics Committee (SOMREC) under registration SOMREC-2024–856. Administrative and departmental clearance were obtained from Mulago ISS clinic and the Clinical Epidemiology Unit (CEU) respectively. A waiver of consent for use of secondary data in the quantitative study was also obtained while informed written consent was obtained from participants of the qualitative study.

## Results

### Socio-demographic, behavioral and clinical characteristics of the study participants

The mean age of the participants was 44 (SD ± 10) years. Of the 390 participants, about two thirds (65.1%, n = 254) of them were female. More than half (55.9%, n = 218) were married and 59.2% (n = 231) were staying outside Kampala. About 4 in 10 (42.8%, n = 167) had primary level as the highest level of education attained. More than three quarters (85%, n = 322) of the participants had no history of alcohol use and 82.6% (n = 322) were employed (Table 1).

**Table 1:** Socio-demographic, behavioral and clinical characteristics of 390 PLHIV that were enrolled on TPT.

Variable	Category	Mean (SD)	n	Percentage
Age (years)		44(10)		
Sex	Male		136	34.9
	Female		254	65.1
Marital Status	Divorced		93	23.8
	Married		218	55.9
	Single		67	17.2
	Widowed		12	3.1
Residence	Kampala		231	59.2
	Outside Kampala		159	40.8
Education Level	Primary		167	42.8
	Secondary		140	35.9
	Tertiary		59	15.1
	No education		24	6.2
Alcohol use	Yes		58	14.9
				
	No		332	85.1
Employment Status	Employed		322	82.6
	Not employed		68	17.4
**Baseline CD4 count (cells/µl)**		444.5(279,642)		
**Baseline viral load**	Undetected		319	81.8
	detectable		71	18.2
**WHO HIV/AIDS clinical stage**	Stage 1		364	93.3
	Stage 2		16	4.1
	Stage 3		8	2.1
	Stage 4		2	0.5
**Co-morbidities**	Hypertension		100	25.6
	Diabetes		1	0.3
	Hypertension & Diabetes		5	1.3
	None		284	72.8
**Hepatitis B test**	Done		39	10.0
	Not done		351	90.0
**TPT regimen**	3HP		131	33.6
	INH		259	66.4
**Type of TPT**	Primary		52	13.3
	Secondary		338	86.7
**ART regimen**	ABC/3TC/DTG		21	5.4
	ABC/3TC/EFV		3	0.8
	AZT/3TC/DTG		7	1.8
	TDF/3TC/DTG		349	89.4
	TDF/3TC/EFV		10	2.6
**ADR history**	Yes		9	2.3
	No		381	97.7

n-sample size, (SD)standard deviation, WHO-World Health Organization, HIV-Human Immune Virus, AIDS-Acquired Immune Deficiency Syndrome, TPT-Tuberculosis preventive treatment, 3HP-rifapentine once weekly for 12 weeks, INH-Isoniazid for six months, ART-Antiretroviral treatment, CD4-Cluster of differentiation4, ABC/3TC/DTG-Abacavir/Lamivudine/Dolutegravir, ABC/3TC/EFV-Abacavir/Lamivudine/Efavirenz, AZT/3TC/DTG-Zidovudine/Lamivudine/Dolutegravir, TDF/3TC/DTG-Tenofovir Disoproxil Fumarate/Lamivudine/Dolutegravir, TDF/3TC/EFV- Tenofovir Disoproxil Fumarate/Lamivudine/ Efavirenz, ADR-Adverse drug reaction.

The participants had a median CD4 count of 444.5 (Q1 = 279, Q3 = 642) cells/ul. Of the 390 participants, 81.8% (n = 319) had undetectable viral loads and 93.3% (n = 364) were in WHO clinical stage one. Almost three quarters (72.8%, n = 284) had no history of comorbidity, 90.0% (n = 351) had no hepatitis B test results and 66.4% (n = 259) had been enrolled on INH. More than three quarters (86.7%, n = 338) of the participants had been enrolled on secondary TPT, 89.4%(n = 349) had been enrolled on TDF/3TC/DTG and 97.7% (n = 381) had no history of adverse drug reaction (ADR) (Table 1).

### Prevalence of non-adherence to hepatotoxicity monitoring

The overall prevalence of non-adherence to hepatotoxicity monitoring was 87.4% (95% Confidence interval (CI): 83.7–90.4). The prevalence of non-adherence to hepatotoxicity monitoring was higher in participants aged 36 years and above (91.9%) as compared to those aged 35 years and below (67.1%) (Table 2).

**Table 2:** Prevalence of non-adherence to hepatotoxicity monitoring among 390 PLHIV who enrolled on TPT.

Variable	Adherence, n (%)	Non-adherence, n (%)	95% CI
Overall non-adherence	49(12.6) *	341(87.4)	83.7-90.4
Age (years)			
≤ 35		47(67.1)	55.2-77.1
>35		294(91.9)	88.3-94.4
Sex			
Male		116(85.3)	78.3-90.3
Female		225(88.6)	84.0-92.0
Marital status			
Married		194(89.0)	84.1-92.5
Widowed		9(75.0)	43.3-92.2
Divorced		77(82.8)	73.7-89.2
Single		61(91.0)	81.3-96.0
Residence			
Kampala		203(87.9	83.0-91.5
Outside Kampala		138(86.8)	80.5-91.3
Education level			
Primary		147(88.0)	82.1-92.2
Secondary		127(90.7)	84.6-94.6
Tertiary		46(78.0)	65.5-86.6
No formal education		21(87.5)	67.0-96.0
Alcohol Use			
No		295(88.9)	85.0-91.8
Yes		46(79.3)	66.8-87.9
Employment status			
Employed		284(88.2)	84.2-91.3
Not employed		57(83.8)	73.0-90.9
WHO HIV/AIDS clinical stage			
Stage 1&2		315(86.5)	82.6-89.7
Stage 3&4		26(100.0)	
History of Co-morbidities*			
None		236(83.1)	78.3-87.0
Yes		105(99.1)	93.5-99.9
Hepatitis B test			
Not done		339(96.6)	94.1-98.1
Done		2(5.1)	1.3-18.7
TPT regimen			
INH		244(94.2)	90.6-96.5
3HP		97(74.0)	65.8-80.9
Type of TPT			
Secondary		337(99.7)	97.9-99.9
Primary		4(7.7)	2.9-19.0
ART regimen			
DTG based		326(86.9)	83.1-90.0
EFV based		15(100.0)	
ADR history			
No		337(88.4)	84.8-91.3
Yes		4(44.4)	16.5-76.4
Baseline HIV VL			
Undetectable		275(86.2)	81.9-89.6
Detectable		66(93.0)	84.0-97.1
CD4 Count			
≥200 cells/µL		292(89.3)	85.4-92.2
<200 cells/µL		49(77.8)	65.7-86.5

*- Adherent patients had only baseline LFTs prior to TPT initiation; none had follow-up LFTs at 3 months or any other time during TPT, (n) Sample size, (%) percentage, CI-Confidence interval, ≤ -Less than or equal to, > -greater than, TPT-Tuberculosis preventive treatment, INH-Isoniazid for six months, 3HP-rifapentine once weekly for 12 weeks.

### Factors associated with non-adherence to hepatotoxicity monitoring

At bivariate analysis age, marital status, education level and alcohol use had p-values less than 0.2 hence were considered for multivariate analysis. At multivariate analysis, the type of TPT was associated with non-adherence to hepatotoxicity monitoring (adjusted prevalence ratio 0.15, 95% Cl (0.04–0.55)). Having Hepatitis B results was a confounder for this association (Table 3).

**Table 3:** Factors associated with non-adherence to hepatotoxicity monitoring among 390 PLHIV that had enrolled on TPT.

Variable	Non-adherent n (%)	Adherent n (%)	cPR (95% CI)	P-value	aPR (95% CI)	P-value
**Age**						
>35	294(91.9)	26(08.1)	1.00			
≤ 35	47(67.1)	23(32.9)	0.73(0.62-0.86)	<0.001		
**Sex**						
Female	225(88.6)	29(11.4)	1.00			
Male	116(85.3)	20(14.7)	0.96 (0.89-1.05)	0.370		
**Marital status**						
Married	194(89.0)	24(11.0)	1.00			
Widowed	9(75.0)	3(25.0)	0.84(0.61-1.17)	0.310		
Divorced	77(82.8)	16(17.2)	0.93(0.84-1.03)	0.173		
Single	61(91.0)	6(09.0)	1.02(0.94-1.12)	0.613		
**Residence**						
Kampala	203(87.9	28(12.1)	1.00			
Outside Kampala	138(86.8)	21(13.2)	0.99(0.91-1.07)	0.753		
**Education level**						
Primary	147(88.0)	20(12.0)	1.00			
Secondary	127(90.7)	13(09.3)	1.03(0.95-1.11)	0.444		
Tertiary	46(78.0)	13(22.0)	0.89(0.76-1.03)	0.106		
No formal education	21(87.5)	3(12.5)	0.99(0.85-1.17)	0.942		
**Alcohol Use**						
No	295(88.9)	37(11.1)	1.00			
Yes	46(79.3)	12(20.7)	0.89(0.78-1.02)	0.104		
**Employment status**						
Employed	284(88.2)	38(11.8)	1.00			
Not employed	57(83.8)	11(16.2)	0.95(0.85-1.06)	0.373		
**WHO HIV/AIDS clinical stage**						
Stage 1&2	315(86.5)	49(13.5)	1.00			
Stage 3&4	26(100.0)	0(00.0)	1.16 (1.11-1.20)	<0.001		
History of Co-morbidities*						
None	236(83.1)	48(16.9)	1.00			
Yes	105(99.1)	1(00.9)	1.19 (1.13-1.26)	<0.001		
**Hepatitis B test**						
Not done	339(96.6)	12(03.4)	1.00		1.00	
Done	2(5.1)	37(94.9)	0.05 (0.01-0.21)	<0.001	0.33 (0.05-2.14)	0.247
**TPT regimen**						
INH	244(94.2)	15(05.8)	1.00			
3HP	97(74.0)	34(26.0)	0.77 (0.71-0.87)	<0.001		
**Type of TPT**						
Secondary	337(99.7)	1(00.3)	1.00		1.00	
Primary	4(7.7)	48(92.3)	0.08 (0.03-0.20)	<0.001	0.15 (0.04-0.55)	0.004
**ART regimen**						
DTG based	326(86.9)	49(13.1)	1.00			
EFV based	15(100.0)	0(00.0)	1.15 (1.11-1.20)	<0.001		
**ADR history**						
No	337(88.4)	44(11.6)	1.00			
Yes	4(44.4)	5(55.6)	0.50 (0.24-1.05)	0.065		
**Baseline HIV VL**						
Undetectable	275(86.2)	44(13.8)	1.00			
Detectable	66(93.0)	5(7.00)	1.08 (1.00-1.17)	0.057		
**CD4 Count**						
≥200 cells/µL	292(89.3)	35(10.7)	1.00			
<200 cells/µL	49(77.8)	14(22.2)	0.87 (0.76-0.99)	0.049		

(n) sample size, (%) percentage, cPR-Crude prevalence ratio, aPR-Adjusted prevalence ratio, CI-Confidence interval, ≤ -Less than or equal to, > - greater than, ≥ -greater than or equal to, < -less than, WHO-World Health Organization, HIV-Human Immune Virus, AIDS-Acquired Immune Deficiency Syndrome, INH-Isoniazid for six months, 3HP-rifapentine once weekly for 12 weeks, CD4-Cluster of differentiation4, TPT-Tuberculosis preventive treatment, ART-Antiretroviral treatment, DTG-Dolutegravir, EFV-Efavirenz, ADR-Adverse drug reaction, HIV-Human immune virus, VL-Viral load, cells/µL-Cells per microliter, History of Co-morbidities* (Hypertension only, diabetes only or hypertension and diabetes).

### Results from the qualitative study

#### Description of the participants.

There were 10 participants of which five were health workers (three medical officers, one clinical officer and one laboratory technician) and five PLHIV that had been enrolled on TPT between January 2022 and December 2023 (Table 4).

**Table 4:** Characteristics of five health workers and five patients that were enrolled for in-depth interviews at Mulago ISS clinic.

Code	Sex	Cadre	Years of experience	Level of education	Occupation	TPT regimen	Date of initiation
HW01	F	MO	5				
HW02	M	MCO	7				
HW03	F	MO	6				
HW04	M	MO	8				
HW05	M	LT	12				
P01	F			Tertiary	social worker	3HP	27/11/2023
P02	F			Secondary	business	INH	02/06/2022
P03	F			None	None	3HP	01/01/2023
P04	M			Primary	Casual worker	INH	01/01/2022
P05	M			Tertiary	Teacher	3HP	27/01/2023

F-Female, M-Male, HW-Health worker, P-patient, MO-Medical officer, MCO-Medical clinical officer, LT-Laboratory technician, INH-Isoniazid for six months, 3HP-rifapentine once weekly for 12 weeks, TPT-Tuberculosis preventive treatment.

### Patients’ and health workers’ perceptions about non-adherence to hepatotoxicity monitoring

Patients’ and health workers’ perceptions were grouped into five constructs of the health belief model. They mainly perceived that hepatotoxicity monitoring is important for proper patient management. They however highlighted some challenges such as knowledge and communication gaps, and financial constraints.

### Theme 1: Perceived susceptibility and severity

#### Sub theme: Awareness of TPT related hepatotoxicity.

Patients demonstrated a clear understanding of the risks associated with TPT and its potential to cause liver toxicity. Many participants were aware that TPT could negatively impact their liver health, and they had been informed by their healthcare providers about these risks. For example, one patient and doctor said,

*“….. the doctor told me I could experience body weakness, yellowing of eyes and that I should return in case I notice these signs….” (* ***P01).***

*“….. we tell you about the drugs, its purpose and the possible side effects……….”* ***(HW01)***.

#### Sub theme: Acknowledgement of serious health risks.

Participants acknowledged the serious health risks posed by TPT-related hepatotoxicity. There was a prevalent fear of experiencing side effects, which underscored the severity of the potential liver damage. Additionally, patients expressed a need to be cautious when undergoing TPT, indicating an understanding of the importance of preventive measures to mitigate these risks. For example, one patient said,

“…….. *we know these drugs can cause liver damage, and if you develop liver damage you are just thrown to Kiruddu hospital so I am always scared*…….” **(*P02).***

### Theme 2: Perceived benefits

#### Recognition of the importance of hepatotoxicity monitoring.

Both clinicians and patients recognized the importance of regular hepatotoxicity monitoring. They appreciated that consistent monitoring could help detect early signs of liver damage, which would allow for timely intervention and prevention of severe complications. This positive attitude towards liver health monitoring is indicative of the perceived benefits of engaging in such health practices. For example, some patients and clinicians said,

*“…….. I have no problem with testing as it’s better to know my health status. I have tested for other health conditions more than once, though never specifically for liver toxicity due to TPT …….” (* ***P01).***

*“………It’s good to test the liver because people tell me that if you are taking many drugs, you need to test yourself…….” (* ***P03).***

*“………. remember treating the liver or kidney is not an easy thing, and it’s expensive. If HIV centers can come up with one voice/resolution and call up the government, we could insist that TPT should not be given without proper liver tests before and after treatment…….” (* ***HW02).***

### Theme 3: Perceived barriers

#### Sub theme 1: Financial constraints.

A significant barrier identified was the financial constraints faced by the health facility and patients. The clinicians affirmed that the health facility lacks funds to implement hepatotoxicity monitoring. They further stressed how patients are unable to meet the costs of LFTs on a private basis. This financial hurdle is a critical impediment to regular monitoring and overall liver health management. For example, a doctor and the laboratory technician said,

*“………….. When funding decreases and we stop doing LFTs, we ask our clients to do these tests privately. It is hectic for them, since many are financially constrained and it somehow affects the whole process…….” (* ***HW03).***

*“……. hepatotoxicity monitoring requires doing some tests, and some of them are expensive and not sponsored here, yet they are required……….”* ***(HW04).***

#### Sub theme 2: Knowledge and communication gaps.

The patients revealed notable gaps in knowledge and communication. Participants reported a lack of understanding about the importance of liver monitoring, which hindered their compliance with recommended health practices. Additionally, difficulties in communication between healthcare providers and patients were mentioned, leading to misunderstandings and a lack of proper guidance regarding hepatotoxicity monitoring. For, example a patient and doctor said,

*“……Doctors need to explain to patients about the importance of testing or assign focal people to do so, as some patients rest in their homes when they are sick because they are not educated about the importance of testing ………” (* ***P03).***

*“………. there is a knowledge gap among patients, and some experience language barrier.* As a doctor, *I have to communicate persuasively, especially when requesting tests that add an extra bill to the patient………”* ***(HW05).***

### Theme 4: Cues to action

#### Triggers to engage in health-promoting behaviors.

Various triggers were identified that motivated clinicians to engage in health-promoting behaviors. Efforts to sensitize patients about the importance of liver monitoring played a crucial role in encouraging proactive health management. Additionally, the use of focal people to bridge information gaps was highlighted as an effective strategy to disseminate information and promote engagement in health monitoring activities. For example, a doctor said,

*“………. some people cannot interpret liver function tests, so education is key. Through CMEs or other approaches, health workers and peers can be taught about hepatotoxicity ……….” (* ***HW05).***

### Theme 5: Self efficacy

#### Confidence in ability to take action.

Health workers expressed confidence in their ability to influence and take charge of patients’ liver health. Efforts to influence policy through research were seen as empowering, providing patients and healthcare providers with a sense of agency. This confidence was reflected in their willingness to advocate for better resources and practices in hepatotoxicity monitoring, aiming to improve overall liver health outcomes. For example, a doctor said,

*“……….. More research is needed, not only on TPT but also on other drugs. If we publish more findings, we can influence policymakers to change the system and respond to our concerns ……….”* ***(HW02)***

## Discussion

This study determined the prevalence, patients’ and health workers’ perceptions, and factors associated with non-adherence to hepatotoxicity monitoring among adult PLHIV receiving TPT. The study revealed a high prevalence of non-adherence to hepatotoxicity monitoring with about 22 in every 25 PLHIV non-adherent to hepatotoxicity monitoring while on TPT. While the WHO guidelines prioritize symptom-based monitoring, MOH strongly recommends performing LFTs for patients on TPT in sentinel sites at baseline, three months after initiation, and as needed based on clinical assessment for early hepatotoxicity detection [2,5]. Non-adherence at this rate is high suggesting that the current practice is not aligning with these recommendations. This implies that there might be an increased risk of un detected liver damage, potentially causing severe health complications to patients [13]. These findings resonate with those in the current qualitative study, which revealed that hepatotoxicity monitoring was foregone for most of the patients due to laboratory stock-outs and, knowledge and communication gaps among patients. The prevalence reported in this study is slightly lower than the prevalence of 95% (630/660) reported in a cross-sectional study done in three tertiary hospitals in Uganda [7]. However, the study by Nanyonga and colleagues was primarily designed to measure the prevalence of suspected IPT-linked ADRs while the primary objective of this study was to measure the prevalence of non-adherence to hepatotoxicity monitoring.

The comparison of the prevalence of non-adherence to hepatotoxicity monitoring in this study with other literature is complicated because most studies report the prevalence or incidence of TPT related hepatotoxicity.

At multivariate analysis, there was no significant association between non-adherence to hepatotoxicity monitoring and demographic, behavioral and most clinical factors. This could be attributed to the fact that hepatotoxicity monitoring was not prioritized depending on any key patient clinical and demographic characteristics. This resonates with the qualitative results which revealed that the laboratory was not able to perform LFTs for patients even when clinically indicated due to recurrent stock-outs of reagents. This problem is not unique to Mulago ISS clinic but has been reported across several Ugandan public health facilities [14]. Furthermore, even when there was room for allowing patients to privately meet these services from outside Mulago ISS clinic, most of them could not due to financial constraints. This implies that without the required hepatotoxicity monitoring, there is a high likelihood of undetected hepatotoxicity as it may be asymptomatic and later progress to severe liver damage [15,16]. This is much possible in patients who are old and/or with advanced HIV as they are at risk of developing hepatotoxicity which would necessitate regular monitoring [17,18].

However, lack of association with some variables like ART regimen, ADR history and WHO clinical stage could also be attributed to the small numbers in some categories of these variables and should therefore be interpreted cautiously. This could have limited the power to detect significant associations. Despite not significant in our study, these are key clinical factors that need to be prioritized as regards to hepatotoxicity monitoring given their relationship with the development of hepatotoxicity. For example, some ART regimens have been reported to be having hepatotoxicity as their side effects [19]. Similarly, TPT regimens like 6H has been reported to pose a high risk of hepatotoxicity [20]. This would require cautious management of these patients as they are enrolled on TPT with regular monitoring for hepatotoxicity for better treatment outcomes.

Results of multivariate analysis show a significant association between type of TPT and non-adherence to hepatotoxicity monitoring. The prevalence of non-adherence to hepatotoxicity monitoring in PLHIV receiving primary TPT was 85% lower than those receiving secondary TPT (PR = 0.15). However, this association might be specific to the operational context of the clinic and its care delivery structure rather than differences in patient or provider behavior. Primary TPT patients at the Mulago ISS clinic are primarily newly enrolled on ART, with whom baseline investigations that include LFTs are done as required by HIV management guidelines. With TPT given three months after ART initiation, it makes patients in this category by default adherent to the baseline requirement for hepatotoxicity monitoring prior to TPT initiation as compared to their counterparts receiving secondary TPT [5]. This insight could inform interventions aimed at improving adherence among patients on secondary TPT by adopting similar baseline monitoring practices. Having hepatitis B test results negatively confounded the association between type of TPT and non-adherence to hepatotoxicity monitoring as the prevalence ratio shifted from 0.08 to 0.15 upon adjusting for having hepatitis B test results. This is because hepatitis B test was related to both type of TPT and non-adherence to hepatotoxicity monitoring since hepatitis B test is one of the tests along with LFTs that were done at baseline and by default the patients who were tested were later enrolled on to primary TPT.

From the in-depth interviews, participants were aware of the negative impact TPT could have on liver health and had been informed by their healthcare providers about these risks. This awareness reflects effective communication between patients and healthcare providers, highlighting the importance of education in managing treatment and monitoring side effects. Such understanding among patients is crucial for fostering adherence to hepatotoxicity monitoring protocols and ensuring timely identification and management of any adverse effects.

Health workers generally perceived that hepatotoxicity monitoring is key and important in effective management of PLHIV on TPT. They further clarified that relying on monitoring for symptoms of liver damage is not enough as it can be easily detected using the laboratory monitoring earlier on before the side effects can even show up. These findings agree with those from a study conducted in Netherland from which 12 in depth interviews were conducted. The study examined factors that influence compliance with guidelines for laboratory testing [21].

Health workers perceived that lack of resources to facilitate the laboratory in providing liver function testing services was a major adherence barrier to hepatotoxicity monitoring. Similarly, patients noted that lack of information and money were barriers. However, some patients might afford the test, but without being informed about its necessity, they are unlikely to pursue it.

The findings are in agreement with those reported in a concurrent explanatory mixed methods that explored perceptions of 12 health workers. The study reported that inadequate viral testing load consumables was one of the barriers of adherence to viral load testing guidelines [22].

There were various triggers motivating clinicians to engage in health-promoting behaviors, alongside efforts to sensitize patients about the importance of liver monitoring, which would significantly enhance proactive health management. These efforts ensure that patients are well-informed and more likely to adhere to recommended monitoring protocols, leading to better health outcomes. Additionally, utilizing focal people to bridge information gaps proves effective in disseminating crucial health information and encouraging patient engagement in monitoring activities. Collectively, these strategies foster a collaborative healthcare environment, optimize resource use, and support the development of targeted interventions and policies to improve liver health management among patients.

### Trustworthiness of the qualitative study

The PI, along with the research assistants who were volunteer clinical officers at Mulago ISS clinic, were able to build rapport with participants. Their familiarity with the clinical setting facilitated open and honest discussions during the in-depth interviews which ensured credibility of the qualitative study.

The research team ensured data accuracy by conducting member checking during interviews, summarizing key points, and asking for confirmation or correction, thereby validating findings and allowing participants to clarify their statements.

Multiple data sources, including patients’ and healthcare workers’ perceptions, were triangulated to enhance the confirmability of the findings. This approach helped to ensure that the conclusions drawn were grounded in the data rather than researcher bias.

### Strength and limitations

The study utilized a mixed methods approach for which the qualitative findings provided valuable insights about the high prevalence of non-adherence to hepatotoxicity monitoring and the underlying reasons.

Being a retrospective study, the current study did not include all potential confounders such as level of income whose data is not captured in patient files and EMR of Mulago ISS clinic. Patients’ level of income would be crucial in the current study as it can determine one’s ability to afford the cost of hepatotoxicity monitoring on a private basis.

Patients were classified as non-adherent when they were not monitored for hepatotoxicity on the three occasions. This could have classified partially non-adherent patients as adherent thus potentially introducing misclassification bias (information bias).

Considering that some variables had zeros in some categories, the study wasn’t adequately powered to detect some associations which could have potentially introduced random error.

Therefore, while the findings of the current study may have limitations in internal validity and generalizability among non-sentinel settings, where laboratory-based hepatotoxicity monitoring is typically guided by clinical symptoms, they still provide valuable insights specific to this setting and other PV sentinel sites.

### Positionality/ standpoint of the researcher

The PI holds a bachelor’s degree in medical laboratory along with experience in HIV care and treatment. This has instilled in him a strong belief in the critical role of diagnostic services in patient management and health outcomes. He acknowledges that his professional bias could have influenced on how he interpreted data related to non-adherence and the perceived value of hepatotoxicity monitoring. Nevertheless, his dedication to improving the health outcomes of PLHIV through the efficient use of diagnostic services drove his interest in this research.

## Conclusions

The prevalence of non-adherence was high. Secondary TPT was significantly associated with non-adherence to hepatotoxicity monitoring. Health care workers acknowledge the importance of hepatotoxicity monitoring but demonstrated implementation challenges due to financial constraints. Patients demonstrated existence of an information gap about the need to be informed more about hepatotoxicity and the need for its monitoring during the course of TPT. The health authorities are encouraged to enhance the implementation of policies that promote regular hepatotoxicity monitoring in PV sentinel sites and, also allocate resources that support these initiatives. ART clinics could also strengthen education programs for patients to raise awareness about hepatotoxicity and need for its monitoring.

## Supporting information

S1 File STROBE-checklist. (PDF)

S2 File Qualitative_transcripts. (DOCX)

S3 File Quantitative_dataset_modified. (XLS)

S4 File Interview Guides. (PDF)

S5 File Quantitative extraction tool. (PDF)
