# Supplementary material for: Prevalence, perceptions and factors associated with non-adherence to hepatotoxicity monitoring among people living with HIV on tuberculosis preventive treatment at Mulago ISS clinic
Source: PLoS One. 2026 Mar 30;21(3):e0345662. doi: 10.1371/journal.pone.0345662 (PMC13035147; doi:10.1371/journal.pone.0345662)
Supplement: S2 File — (DOCX) [file pone.0345662.s002.docx]

**P01**

**Interviewer: What do you understand by liver effects/hepatotoxicity due to TPT?**

**P01**: No, what I know those drugs most times, our body responds differently to this drug because some other people get effects and others do not get effects. For me I took the drugs and and I did not get any effect, but some other people took those drugs and they got some effects and they told me the liver becomes weak.

**Interviewer: What did the doctor tell you about the TPT drug before giving you these drugs?**

**P01**: The doctor told me I could get some changes like body weakness, yellowing of eyes and when I get the signs, they told me to come back. Actually, I became too thin when I started taking these drugs and the doctor told me this effect.

**Interviewer: Were you convinced by the Doctors medical information about TPT and felt like you had received enough information about the drug.**

**P01**: The truth they never taught me well about that drug and I was convinced by the word prevention is better than cure and I kept on taking the drug.

**Interviewer: What do you think was not explained well to you about the drug?**

**P01**: For us who have been taking drugs, like when I started my HIV drugs, I started with Septrin and when I changed to other drugs, they taught me well but for this specific drug they told me a few signs and they said when I see them, I can come back to the facility.

**Interviewer: What do you think about testing the liver health in regards effects that could be caused by TPT drugs?**

**P01**: Doctors. Me I have no problem so long as its part of my health and it’s better to know my health status and I have tested more than one time for other health problems to know my health though I have never tested specifically to know my liver health due to TPT.

**Interviewer: What could be the barriers that could hinder you from testing liver health?**

**P01**: The fact that I am already sick scares me to test and know other complication would bring me some pressure.

**Interviewer: What ideas do you have for us to help improve the compliance to hepatotoxicity testing and monitoring?**

**P01**: You doctor you need to sit down people and explain to the people the importance of testing or put out focal people to do the teaching about testing because sometimes people rest in their homes when they are sick but because they are not educated about the importance of testing then they cannot test. There should be focal people in villages who could keep the confidentiality and these people would help incase us patients want to share about our health.

**Interviewer: Do you anything to share with me.**

**P01**: They did well to bring those drugs because if we get TB and the same time we have HIV, then it would be the worst moment. They should actually bring more drugs. The truth I was not convinced initially because I really took those drugs and I did not even know the outcome but I continuously took the drugs and I was really sad with the doctor but when the doctor explained to me, I became convinced and understood because the situation I had of thinness was just for that moment and I was sure if I get TB, It would not treat me so badly like someone who had not taken the drug. When it comes to testing, I have no problem because I want to always know my health and the liver has no spare (replacement) so I need health and I will always test.

**P02**

**Interviewer: Why do you understand by hepatotoxicity due to TPT?**

**P02**: I remember I swallowed those drugs once last year and it was in May. They taught me the side effects and told me that I feel any side effect I should come and see the doctor and they gave me the contacts.

**Interviewer: What did the doctor communicate about the side effects?**

**P02**: The doctor told me there will be change in Urine colour, body rash and yellowing of eyes and dizziness. The doctor said i need to take enough water and this can help to reduce change in urine colour and the colour returns to normal.

**Interviewer: Did you really feel that you really understood what the doctor told you?**

**P02**: Yes, I felt that I had understood, but you know when someone tells you you are going to get rash and also yellowing of eyes and you have been taking your HIV tabs without all these issues, the truth is I got scared because when people start seeing you with rashes, they will start thinking they are due to your HIV status. At the start I even got scared to swallow the tablets coz I was thinking if I die after taking them, but the doctor has convinced me well and told me if I see any side effects I come back to the hospital immediately.

**Interviewer: What did you fail to get convinced of in the Doctors explanation?**

**P02**: I was not happy the fact that the doctors know that we are HIV patients, and they give us drugs that have side effects, and they can cause rash or yellowing of eyes to us. Also, the fact that they did not even first tests us if you have high blood pressure or Diabetes and even we have other drugs for those co-morbidities. You even worry that those drugs might even rise your blood pressure and you wake up one day with a rush. I got even scared and felt as if there was a rise in my pressure value even and another thing I was also scared that when you get too sick, they even give you a referral and you find yourself in another hospital like Kiruddu hospital

**Interviewer: What is your opinion of this medication of TPT?**

**P02**: The facts that I was on HIV drug for so long, Testing the liver is not bad but it scares because you start thinking that you are HIV drugs and now you add another drugs which is also damages the liver and then when you get liver complications then the Doctor might say its because of the HIV drugs and he/she doesn’t agree that it might be due to the TPT drugs and most moments its such situation that scares.

**Interviewer: What challenges would hinder you to test for hepatotoxicity?**

**P02**: The main problem is the worry I get when I think about the status of my liver because you start thinking now if your liver is found damaged there is even no repair, so you rather remain without knowing your status. Also, even you know even the drug you are using can cause liver damage and we even know that if you are found of liver failure you are just thrown to Kiruddu hospital so I am always scared.

**Interviewer: Is there anything you would like to share with me about the management of hepatotoxicity.**

**P02**: Doctor, what I request is before they bring these drugs, they should first explain well about the drugs, and you see if they really prevent or cure. You see like the TPT drugs they told us it prevents but it doesn’t not cure and now you see that you can even get TB when you are taking those drugs and now you are already suffering with the drugs side effects but it is not even helping you at all because you can even get TB when taking TPT drug even sometimes you find you are already on blood pressure and diabetic drugs and all this pill burden. Secondly before you give us these drugs first do some checkups like for diabetes and blood pressure and you see that we are on these other drugs, and you don’t give us this other drugs because the drugs are many and even get tired of taking because of the pill burden. Thirdly, most times you tell us some of these drugs are for clinical trials and they always have much side effects, so most time we need doctors to do a follow up incase of any side effects so that we cannot struggle in case of any problem in the place they sent us. What surprises me that drug is also likely to be taken daily like HIV drugs because the doctor told me that you still the take the drug after a certain period and I thought you would take the drugs once and finish. Now we get scared that now if last time I survived the effects now what about this time when I take what will happen and you know our life weakens every day.

**P03**

**Interviewer: The liver problems you know when you take these drugs**

**P03**: Mainly yellow eyes, yellow skin, you loose appetite

**Interviewer: How do you get to know about these problems**

**P03**:I heard some information through reading from internet.

**Interviewer: Did you get any medical information when you were giving these drugs**

**P03**:You might get Nausea, you might loss appetite and get itching on the skin

**Intervier:Do you feel the medical Information was enough from the doctor?**

**P03**: No, it wasn’t because they did not first check if our liver is ok or not or if our we are eligible for the drug and me, I thought they would first test us. Me my concern is about the test and they could first check if I am eligible for this drug and now they just gave us the drug.

**Interviewer: When you went home and took the drugs, are there other signs you saw on yourself that showed that maybe you were not given enough information.**

**P03**:Yes, I felt general body weakness, and they only told us about rashes, itching and nausea, and they never told us about general body weakness

**Interviewer: What do you think about checking for liver problems due to TPT.**

**P03**: They have to first check if someone is eligible for that drug before even giving the drug.

**Intervier: What could be the problem that would stop you from checking liver health as suggested by the doctor.**

**P03**: Me I have no problem with checking first before they give me the drug. The test is expensive and I might not afford the test.

**Interviewer: What are your ideas for making sure patient for monitoring for liver health?**

**P03**: Of course, no one can refuse, and most of the patients can not afford and if there is an offer then the patients can not refuse. The facility should put the test as a mandatory that when you are changed to another drug it’s mandatory to test.

**Interviewer: Is there anything you would wish to share with me about the TPT and liver monitoring?**

**P03**: Nothing much and the little I can say is they have to first check the person before switching them to another drug.

**PO4**

**Interviewer:**Hmmm, we don't want to use your name ahh so that it doesn’t appear in the what in the recording . so that if someone may be hears it and knows did so and so, I don't know whether it's ok with giving you the code like may be calling you P04 , we can call P04 eeh Ok P04so we shall have to refer you as a P04 eeh going forward . so i want to remind you that you have to feel free to join the study and if you feel uncomfortable at any point you might request us to stop its entirely voluntary and aah you need to note that aah the record the interview will be recorded and aah to make sure we don't miss any important issue and only the research team can listen to the recordings and they will be aah and they will keep your information prevate ,

**Interviewer:** so during the interview you can interrupt me ,you can ask me any question that you feel like aaah

. so like i told you i have already introduced ourselves here am Kevin aah she's Peace and sh’s ketra they're all part of the study so …..

**P04**- so the study does not aaah dose not involve any other thing about apart from those things your asking ,

**Intervieweer:**:- yeah this this is the only part where you will need to participate you will not have,

**P04**:-how about the medicine ,

**Intervieweer:** :- the medicine like giving you the medicine

**P04**- No No the questions about the medicine

**Intervieweer**:- Hmmm yeah so and i will start and i will request you to speak alittle bit louder so that i can be able to or you can push closer lf you dont mind . so aaah you kindly ,kindly tell me **in your own words what do you understand ahh about liver problems it can happen because TB prevention medicine**

**P04:** may be aah wounds on the liver , swelling ,may be change in size or ,

**Interviewer:** - it is ok it is ok , so it tells that you have something that you know

**P04**- yes ,

**Interviewer** - about the liver problems so in this case aah we are interested aaah to understand to understant what understand by the word liver problems when someones tells you that what comes to your understanding

**P04:** - me what comes to my minds is aah, the failure of the liver and the causes of the failure of the liver are very many as i have told you may be it can develope some wounds, Hmm it can change in size ,

**Interviewer:** true , true

**P04**: it can change in size according to the medication you are taking

**Interviewer:** true,

**P04** it can diminish or it can become small

**Interviewer:** true

**P04** or it can have some small small swellings

**P04** so those are all liver problems

**Interviewer:** hmm , ok its ok it is perfectly well said and now in this case aaah **what did they tell you about any possible bad reactions you may have from the TB prevention medicines**,

**Interviewer**:- may be in the ,from the time you were given that medicine

**P04** :- the doctor just give me and told me that is prevention

medicine and he told me to go and buy some supliments and of which the supliments hmm so ,to the clincs i went to in my area so i decided to to stop in the meddle Kevin hmm with the medicen becouse i had no supliments kevin hmm cn if you

may be what ment the doctor refer to the other supliments they my be nutrolizing kevin hmmso thats what made me stop the medication

**Interviewer** hmm , so ahh they didn’t tell you about ahh any possible bad reaction that you could get cn hmm ,

**Interviewer:** so it wasn’t part of the discussion

**P04** it wasn’t part hmm ,

**Interviewer:**ok so it is ok , it ok the good thing you were given the medicine and advised to get the supliments so thats what they told you about ahh the supliments that thy will help you nutrolize thats what you think cn yeah its what me i think . kevin hmm, CN so if the doctor tells you may be your getting this medicine of ART plus septrin importance of septrin besides the ART

**Interviewer:** so if you take this one alone and yet he has recommended you to take with the other one, that means you going off the truck so that's what made me stop because there was no access

**Interviewer:** hmm, it is ok so aaah do you feel like your are adequately explained to by your clinician about the TB prevention medicine and its side effects

the ones you had

**P04:** i didn’t hear of any side effects the ones i had of an importance that is, that is preventing TB **Interviewer:** hmm ,ok , so the side effects are aah are not part of what you discussed about the medicine CN there not part , so what do you think about checking for liver problems causing by TB prevention medicine

**P04:** I personally or,

**Interviewer:** you what would think is it something that is done , beccouse ahh something that is done to monitor you on whether the liver is ahh

**P04** for me i checked actually may be i checked before the treatment because first of all my skin rash some people would say maybe the liver is not functioning well i went for check up and they told me the liver is normal, so just slightly they told me do not take fatty things and may be limit your sugars , thats what they told me, so that it can stablize,. So it was yeah any way i dont know according to the results you know drs writtings you can not understand well **Interviewer:** so in your opinion ahh what would you talk about that practice of knowing how the liver is working or is it something that you feel is ok like you have to know or you shoudn’t know i don’t know

**P04:** I think it is every ones responsibility to know every thing, even if it is the head paining you , you can go for a scan, may be your skull will have got a problem if your sick and you dont want to go, so it getting , getting ahh knowing about your parts inside it is very important because you may get a problem when you dont know so its mainly only that apart from ignorance its porverty but every one would have gone for medical check up ,

**Interviewer:** hmm ok so that is perfectly said eeh ,ahh what do you think aah i think this is what we have finished . What could be the problems that may stop you from checking your liver heath

**P04** as suggested, that one i have already told you , ok the third thing would have been fear, some people fear to go that the doctor is going to give me some negative information kevin hmm, cn that one is another attitude that makes people not to go for checkup, another thing is lack of enough funds. kevin hmm true, CN people don’t have money your going to go to check you and someone i going to say i need some 200k to scan you and give you the results some one is going to say 200k is the food for my family , cn i will not go kevin hmm another thing is ignorance , you get sick you may be have body weakness you don't have to go to the doctor you need these pain killers u leave just , just because you are ignorant about your life, yeah i think now from what you have said aah aah is ahh nga poverty , mabe ignorance and fear eeh CN hmm ,

**Interviewer:** yeah true , so what , what are your ideas eeeh for making sure that patients follow advise for liver heath checks

**P04** what are my?

**Interviewer:** what are your ideas , what would you suggest to make sure that ahh people follow liver heath checks . so i was asking you what would you suggest making sure that patients ahh follow those ahh follow the liver health checks

**P04** the advise to do those liver heathy checks. P04 its ,its may be bringing knowledge to people, P04 i don't know apart from the heart another part very important part in the body is the liver hmm,P04 because transplanting it is very expensive better than treating it so is is just sensitizing people about the importance of ..

**Interviewer:** the relevancy of of aah checking for the liver . so you feel if you may be aah given enough information aahh about aah the liver how important it is ,, kevin and may be the likely could of it being ahh affected by the medicine cn hmm , kevin you feel some how ,some how people would be better, patients will be encouraged to do the checking , CN yeah but any way there is nothing with an advantage with out a disadvantage ,cn may be what is best is to encourage every one to check so if you tell some one that if you take camadol , ur treating the head but ur

damaging ur intestines

**P04** again that person will not treat the head and will end up running mad so the only thing is senstizing telling them that its very

important hmm, cn , now for example me starting for a heathy check up apart from being acount full of may money , know i value my life , because i think that every minute that passes it me counts it kevin true ,CN so every one just needs to knw hmm, cn importance of being with a health liver

**Interviewer:** hmm , so is there any thing you would wish to share with me about ahh TB prevention medicine or liver heath checks or any other suggestions

.**P04:** the only problem may be what i would share with you is the truth that some medicines , some supliments of the TB , with TB treatment they are not every where atleast i would have been accesible cn may be when you visit some one who is green ,about the side effects now like me who is not sensitized, and just take and leave the other supliment , because you will say let me take this one , hmm , so its best either may be when every thing is approchable , cn you say we are giving you this one but you can get this from there,

**P04** so u give some directions ,

**Interviewer:** so ur challange during that period aah is u weren’t able to access the cn i was not able to access , able to access the supliments ,cn supliments ,

**Interviewer:** and aah so what, any other thing about the side effects aah for the liver about ,cn hmm, kevin about liver checks , the checking eeh is there any thing you would wish to to share with us ,

**P04** may be i wanted is ok may be let me ask may be a question, is there a way of neutralizing that medicine to decrease onthe side effects as suggested as written there ,

**Interviewer:** hmmm so in this case aah this monitoring it helps us to knw how the liver is functioning like you have told me so incase there is any thing it is easy for us to do what to knw when its still early and aah may be we can change the treatment , give you some other treatment or stop that treatment so that

**P04:** the truth is that me when i was taking the medicine, i was feeling headache and i said that may be since i didn’t get the other medicine the doctor told me to get k, so let me stop it , so it didn;t suddenly come that aahahh i should not take the medicine , as i was taking the medicine may be i took it for some 2 weeks and then the 3rd week the head started paining me i said let me first leave it , until because i was and i was to come here and wednesday this week , i said let me come early but i didn;t see the doctor there i wanted to see when i went for viral check up

, hmm,OK, its Ok .THANK YOU.

**P03**

**Interviewer: How do you understand liver disease in relation to TPT?**

I don’t know because if the doctor gives you a drug he/she knows already the use of the drug and what the drug can prevent and for me I don’t know so I follow the doctor suggestions because it’s the doctor who knows. If he tells me to take the drugs I will take before I can not refuse the doctors prescriptions because I expect that the doctor studied knows more and also I can not come to the medical facility and I explain my medical condition and the doctor gives me medication and I refuse to take them because I can not prescribe my own medication because it’s the doctor who knows.

**Interviewer: Do you think we can liver damage when we take TPT drugs?**

**P03:** I am not too sure but I know it might happen.

**Interviewer: Did they provide medical information?**

**P03:** They told me I can get dizziness and vomiting and he said if I get the problems/side effects then I can come back to the facility and they change the medication or get for you medication

**Interviewer: Do you think the Doctor gave you enough medical education?**

**P03:** The doctor asked me whether I have taken TB drugs, he told me there is astudy that we have got where they have brought TB drugs for people with HIV because they can easily get TB but he told me that the drug can present some side effects and if I get those side effects I come back to the hospital and the doctor said things like skin rash, dizziness and vomiting. I could not be hesitant because the doctor had told me to take the drug.

**Interviewer: Do you think its good to test for hepatotoxicity?**

**P03:** Its good to test the liver cause people tell me that if you are taking many drugs you need to test yourself

**Interviewer: What challenges/limitation can hinder you from coming for these tests.**

**P03:** You find if they add you more drugs they may bring more effects to me.

**Interviewer : What suggestion do you have for us in improving hepatotoxicity monitoring?**

**P03:** You have to provide medical education to the patients and even remind the patients to take come for the drugs.

Questions from P03

**P03: Why have they brought this study?**

**Interviewer:** If the people who manufactured this drug directed us to test for liver disease because this drug might cause a problem but the drug is good.

**P03: If you are found sick with liver damage, can you heal?**

**Interviewer:** If we get to know about the disease early, we can prevent the disease.

**P03: What could be the signs of liver damage?**

**Interviewer**: Swelling of the stomach, yellowing of eyes and skin rash but they show later when you already have liver damage

**P03: Do you get free treatment if you are found with liver damage?**

**Interviewer**: It depends, If you find a study with liver damage treatment it can provide the free treatment but also other facilities like Mulago can provide treatment.

**P03: Can I get treatment if I get sick with this medication used in the study?**

**Interviewer**: If the study shows that the patient side effects will be catered for then the company carrying out the research can provide the treatment so always ask these questions when participating in such studies.

**HW03**,

**Interviewer: What are the key points you emphasize to your parents before you enrol them for TPT**

**HW03:** Just to take you back all People leaving with HIV, we make sure they take TPT during a certain point during their treatment encounters and we do that usually through routine screening for those eligible for TPT by looking at their liver function tests, ruling out NCDs like hypertension and DM that may cause symptoms that hinder them from taking TPT, take note of their duration on ART because we see that we give this TPT every 3 months after their attenuation and for those known HIV patients who are known to be on ART, we give them TPT every 2 years. Our main emphasis is on ruling out eligibility criteria on identifying these clients why they need this TPT therapy as we know the TB burden is high in Uganda and we are getting TB patients every hour and then and that’s why we emphasize health education to every client that we receive here at ISS clinic to rule out their eligibility to receive TPT

**Interviewer: In the discussion with you clients, how do you educate your clients when enrolling these clients especially hepatotoxicity and monitoring?**

**HW03:** Thank you for that question, so usually we educate them before TPT and they have to see a counsellor to educate them more before enrolling for TPT and from the counsellors and they come to clinicians and they are asked some social economic questions like do they take alcohol, are they on other TB drugs. But specifically, about hepatotoxicity, usually we have to do LFTS to rule out their eligibility and we educate them about symptoms that show dysfunction of the liver like if they come up with yellow eyes, severe hypochondriac pains and we always emphasize to report back to the facility in case of any symptoms.

**Interviewer: Are there specific barriers you face in sending this message to your patients?**

**HW03:** Some of the barriers are at times you know with fluctuation in funding, and these are donor funded projects, there are not funds to facilitate these tests so it will affect our process for screening for eligibility and also assess our ability to monitor their LFTs to rule out hepatotoxicity. Another thing I can talk about, at times language barrier in terms of some clients may not understand well the question related to liver function and they might give false answers and also sometimes clients may not feel comfortable to disclose their alcohol history and sometimes they lie and mislead us in ruling out their history for alcohol.

**Interviewer: What could the reason for patients not complying with hepatotoxicity monitoring?**

**HW03:** As I said at time in moments when we lose all the funding or funding decrease and we stop doing LFTs, we now ask our clients to do these tests privately as you know its hectic for them and some of them are financially constrained and they don’t have the funds and its some how affects the whole process. Another thing is non-disclosure of dishonest to their alcohol history, we usually emphasize more peer to peer counselling and those are the gaps we have at ISS

**Interviewer: Are there limitation that clinicians encounter through the systems of facility that can hinder them in monitoring hepatotoxicity?**

**HW03:** I can say as I said we run out of funds to run the tests like LFTs due to funding and the stocks of TB preventive therapy. But its usually inability to do these routine tests because we know that we need to do the LFTs before starting TPT, 3 months after starting TPT and after every six months for those suspicious clients that encounter side effects. The test is meant for every client and if the facility can provide funds then all tests can be done.

**Interviewer: How can healthcare systems be improved to enhance compliance to hepatotoxicity monitoring?**

**HW03:** I think through soliciting and looking for further funds to run these tests and through aggressive health education to these clients to limit them from using herbal or these other substances like alcohol to prevent from using those substance along side TPT. So usually we streamline that through detailed health education to our clients every morning and we condemn them to use any other substance like alcohol alongside TPT.

**Interviewer: Is there anything you would wish to share with me about TPT or any suggestions?**

**HW03**: Ok, I think I would emphasize the need for such investigation for liver function in regards to TPT initiation and also emphasize that we really need TB preventive therapy as we know the TB burden currently in Uganda and we also a suggestion if there is a way to always monitor such clients with close monitoring with a focal person and detailed emphasis on follow up calls, calling these clients or checking on them atleast 4-6 weeks to rule out these side effects and they would managed earlier. Emphasis on health education by clinicians and other multidisciplinary parties to rule out adherence to these drugs and limit the use of these substances like alcohol, herbals and any other substances.

**Interviewer: Do you think the information you give to the clients is convincing?**

**HW03:** Apparently, all our clients at ISS are usually talked to through our health educators to always listen to the clinician and always follow up their advice and counsel. I am certain if all the clinicians are given the right information about TPT and its well passed to clients, it would give out a good outcome as regards to TPT uptake and hepatotoxicity monitoring

**HW02**.

**Interviewer: What are the key points that you emphasize to your patients before you enrol them on TPT?**

**HW02:** First of all, we educated them about the side effects and the importance of the drug. Remember this is ISS and their immunity is low, and they are able to easily get TB in the community so we emphasize them to take TPT. We just give me a second thought and its voluntary so if they are not ready in the first visit then we educate them again in the second visit.

**Interviewer: How do you educate your patients about TPT, potential risks and the importance of hepatotoxicity monitoring?**

**HW02:** As TPT is like any other drugs, our patients have the right to know the side effects and how know how they can manage them. Some patients when you talk about the side effects, they become scared but if they are not manageable at home, we tell them to come back immediately to the facility so that we see how we can help them. As I told you in our medical education, we tell them about the potential risks, and we encourage to get in touch with the medical facility incase of any challenge. We do tell them about the risks like to the liver but there are indicators that can show liver problem and we tell them about these indicators like yellowing of eyes and we tell them these indicators. On monitoring, it’s their own benefits and even when we are initiating someone on ARVs we tell them the effects and benefits.

**Interviewer: What are the barriers you face in conveying this information.**

**HW02:** Of course, this is not easy information to tell because you are now telling them it will damage the liver and most cases every time you introduce any new medicine, the ideology of the patients is you are going to finish them. A lot of rumours in the community where the patients have a perspective when the government introduces a new drug, they think the whites want to finish them.

**Interviewer: What could be the reason for patient not complying with hepatotoxicity monitoring?**

**HW02:** The patients are illiterate and when you give some other medication, the just leave the medication there and they don’t take the medication. Even the toxicity talk has to be a routine in the daily medical education, and this could maybe help in compliance and currently this talk is not well captured in the education and even because of high volumes of patients, the counsellors might not really talk more about this information. There is a need to integrate this information in the daily medical education.

**Interviewer: What could be the system based challenges?**

**HW02:** Of course, there are some tests we cannot do because of some reagents and when you tell the patient to pay some money, the patient will say doctor we don’t have and even now the government needs numbers and then because we are given target for those drugs, we end up giving those drugs even without carrying out the tests because we need to report the numbers. If you have the funds, then everything is fine.

**Interviewer: How can healthcare systems be improved to enhance compliance to hepatotoxicity monitoring.**

**HW02:** It’s not the facility, the facility bases on the funds and if the government or funder notice that the TPT drug requires monitoring, they should also provide other necessary things. Not only giving numbers and when they improve that, the things will go well. I cannot say the patient should have money and they are poor, and you cannot tell the patients to do LFTs. Some of them even come here when they are hungry and now you tell them to buy the drugs. I think the cry goes to the government, as they demand for numbers let them provide funds to carry out other tests.

**Interviewer: Anything you would like to share with me about TPT**

**HW02:** Yes, TPT is really a nice therapy to our patients, and they need it though we need to style up how we give our drug. Despite the need for numbers, we need to consider our patients because these are humans, and they have people to care of and if we are not considerate, they are going to end up crying. Remember treating the liver or kidney is not an easy thing and its expensive and if we can avoid that and if the HIV centres can come up with one voice/resolution and call up the government that they tell them we appreciate that you give us medicine but we shall not give it and we give strong reason that we cannot give the medicine without testing because we need to do the tests before and after taking the drugs, but we don’t have that capacity. More research is needed in this area not only TPT but also other drugs. Instead of doing viral load and its for free. I think the government or researchers should come on board and I think if we do more research, we can influence policy makers to change the system. The research like this one is good and we get more research on this matter and we have more papers out then it could help to open up to policy makers and maybe our cry could be answered.

**HW05**

**Interviewer: Key points you emphasize your patients before you enrol them on TPT.**

**HW05:** You have to understand that TPT is TB preventive, its for prevention of TB in patients with HIV, It doesn’t mean that the patients will get HIV but by giving TPT we want to reduce their chance of acquiring TB. We usually tell them its for their benefit the fact that they are living with HIV, they are compromised so we want to put in place measures that help reduce the risk of the opportunistic diseases that come with HIV and TB is among them so we try to emphasize the role of TPT, the regularities and you also tell them the drugs TPT comprises and mention that TPT is TB preventive therapy and we give it out for 2-3 years to try to reduce the risk of getting TB since they are immuno-compromised and of course we start educating them on TPT.

**Interviewer: How do you educate your patients about TPT, potential risk of acquiring hepatotoxicity and the importance of monitoring?**

**HW05:** So, what we usually do, I introduce them to what TPT means, TPT is TB preventive therapy and its swallowed to try and reduce the risk of acquiring TB especially in people that are immune compromised and these HIV positive people and I educate them about its importance and they give them a reference that there different types of TPT depending on the contraindications. The commons ones we are using are the 3HP, so that means that 3HP has two drugs Isoniazid and rifampicin is taken monthly and it’s started weekly for 3 months.

So, you tell them that they are 3 pills once a week for 3 months and you tell them about the potential side effects that are known. The commons ones and for them they don’t understand hepatotoxicity but of course you tell them what signs that can show hepatotoxicity like abdominal pain and yellowing of the eyes are these are some of the common signs. You tell them when you see the eyes are to yellow you come back. You also tell them to drink a lot of water after taking the drugs because its deals with the metabolism of the drug. So, you basically tell them about the symptoms that they might experience and tell them to report back to you. Of course, you tell them about the drugs and tell them they might experience some side effects though not all of them can experience the symptoms.

On importance of monitoring, so long as you tell them that the drug has side effects, they be on the lookout and tell them to come back in case of any side effect.

**Are there specific barriers that you face in conveying this message to your patients?**

**HW05:** Yes, the first one some patients think that when you tell them about TPT they have TB and for you you have always realized that they might get TB and they always ask “Musawo am I going to get to get TB” and they already have that bias and maybe people do not know about TPT and the fact that they do not know about it, they are already biased and they will still ask “Musawo are you going to treat me because I have TB” So majorly there is knowledge gap among patients. Secondly, patient may experience language barriers and as a musawo, you have to have a convincing tongue because you are adding a patient extra bill. Imagine someone has no TB and you are convincing them to take drugs and someone will ask you “am I taking this drug”. The fact that they are asymptomatic, they will be hesitant. Another challenge you find other than the hesitance is the pill burden and they will complain that I already have other drugs and you are adding me more. With the 3HP since its weekly, the acceptance will be easy however if you put someone to isoniazid daily, it will be hard. The other barrier while conveying this message, someone might be in a hurry and you are introducing a drug that is important and now you are introducing a drug and they are in a hurry and they have been in a long line and they are already agitated and they say I will see the drug when I come back next time.

**In your opinion, what could be your reason in patients not complying with hepatotoxicity monitoring?**

**HW05:** I think the patients do not know what hepatotoxicity means and its us medics that know what it is and imagine if people don’t know what it is, so there is a knowledge gap and ignorance among people about hepatotoxicity and for complying incase you provide the drug without conveying enough knowledge to the patients and you dispense, they will not know because you will even see them after 3 months unless they get serious side effects for them to come back. So, of them are given multi drugs and you will not see them for six months, but you have given them TPT, so if you don’t deliver your message well or don’t take time to educate them well about the symptoms of hepatotoxicity they remember because you can’t monitor what you don’t know. The major reason is because sometimes patients can be many, and you don’t get much time to educate them. With the little information about the drug, sometimes some patients will not even take the drug even after confirming to you that they will take the drug just to excite you.

**Are there complications that clinicians encounter in implementing monitoring for hepatotoxicity due to TPT.**

**HW05:** Yes, the fact that we are in a resource limited country. Before you initiate someone on a drug that can cause side effect on someone organ like the liver, you must do the baseline tests and sometimes you don’t have resources to do the baseline tests. Even after initiating the drugs, you might not be able to do the routine monitoring because you don’t have the resources, you don’t have the money to do the lab tests. You even know what is supposed to be done but your hands are tied, and you can do the monitoring and in our setting you might fail to do the lab tests are required in monitoring. You just give the drug and wait for the side effects and that when they could come back and you try to do the tests and still your hands are tied and you cannot do the tests.

**How can you enhance compliance to hepatotoxicity monitoring?**

**HW05:** The first one is to equip people with knowledge about hepatotoxicity and what are the signs and symptoms of hepatotoxicity, what tests do you do when monitoring hepatotoxicity and how do you interpret those results and some people can not even interpret liver function tests so the first thing is to educate people. If people have the knowledge and you tell people if you such things points to hepatotoxicity, so people are now knowledgeable. You could conduct CMEs or any other ways of delivering knowledge to people giving these drugs whether they are health workers or peer and teach them about hepatotoxicity and then provide equipment and resources and basis of monitoring the patients. Put in place an SOP in how we can monitor and if those resources are there, people can comply but what happens the resources are not there even when you want to do something. So put in places measures and access to labs and then put there someone in charge of monitoring and we know that are is someone we know that incase of hepatotoxicity he is the focal person and person will make sure he mobilises people.

**Anything you would wish to share with me about hepatotoxicity monitoring.**

**HW05:** What needs to be done, its goes back to patients, the facts that you are adding pills to them are they are already taking pills, so a lot of emphasis has be done either at triage or like CMEs to these patients because they are one going to take the drugs to ensure that accepting the preventive therapy is not hard and if the patients come to you when they know the role they wont be hesitant. The challenge comes when you meet a patient, and they don’t know even what TPT is and maybe every morning educate the patients about TPT and I think this goes back to us teaching patients about TPT and the gospel about preventive measures has to be preached. Other than about monitoring put the focal person in place.

**HW04**,

**Interviewer: As a lab personnel, what do you understand by the term hepatotoxicity monitoring of TPT among HIV patients?**

**HW04:** Hepatotoxicity monitoring is conducted to see the surveillance and whether the liver is working vs the level of drugs being used for TPT. Its usually done to monitor on the progress of the drug and how the liver is trying to break the drug and there are tests that are conducted monitor that.

**Interviewer: How often do you receive the patients?**

**HW04:** We don’t have get forms specifically hepatotoxicity testing, but we always get forms for liver function tests, and I think hepatotoxicity testing falls here and it’s an often test which is requested since people taking TPT are on other medications for ART and these medications might not be gentle for the liver.

**Interviewer: Are there any trends or patterns in compliance with sample submission?**

**HW04:** After explanation of why we are conducting the tests, most patients tend to comply through the clear guidance from counsellors though there are situations when some people are hesitant with pricks and they try to resist but with time they comply after explanations.

**Interviewer: What challenges if any you encounter in ensuring accuracy in timely monitoring?**

**HW04:** As normal challenges are there though not so many but to some extent, usually monitor requiring doing some tests and some of them are expensive and some are not sponsored here and they are required and most of them go for them on private basis or if there is a waiver, you find all of them take them to Mulago. It becomes hard for patients who can not afford them on private basis, and it becomes hard for us to deliver because the turnaround time of Mulago doesn’t support as the results which were supposed to be used in real time are used later. And you this affects the extent to which the liver is damaged.

**Interviewer: Anyways suggestions for improvements overcome these challenges.**

**HW04:** Some are applicable and others are not applicable, maybe if there could be a way to get on point machines which could help in conducting those tests at the facility could help because patients come once in a while for example if a patient is given a TSA and comes after 3 months and they have a liver that is not functioning well and doctors assess the liver. Having an on point machine would work well or having a place where the tests could be for free, and patients be prioritised could work and having a focal person in that area to prioritize patients would be more easier.

**Interviewer: How do you communicate results and any relevant information to other health workers?**

**HW04:** All the tests that are carried out in the lab are returned to the clinicians for proper management of patients and we always ensure we ensure turnaround time and make sure the results are delivered timely and robustly. We usually communicate them after thorough analysis. We take samples to clinicians on time. Our communication is through clinicians and its from the clinicians to other parties.

**Interviewer: How can collaboration between laboratory personnel, clinicians and patients be improved to ensure compliance with hepatotoxicity monitoring?**

**HW04:** The compliance already exists, may be to strengthen it through radio communication and CMEs to show how serious hepatotoxicity is and involving all parties since it’s a team. You should not exclude any other team member or consider lab technician alone or clinicians alone, so there is need for clear flow of information and work as a team. Another thing to be done between the clinicians and the lab is to prioritize the tests and show them as emergency and they be given priority.

**HW01**

**Interviewer: What are the key points you emphasize to your patients before you enrol them on TPT?**

**HW01:** Firstly we go through the criteria, they have to be 2 years or above, they have to be on ART for more than 3 months if they are new, they don’t have to be with symptoms of TB or anything suggestive of TB and those are the first things we look at so if you pass the criteria, we tell you about the drugs, its purpose and the possible side effects. Ideally, we are supposed to tell you information about all available drugs but we always tell them on VHP because it has been the available in stock and we would tell you about that one, but we had more than one in stock like when we had QT and INH. We were using INH (Isoniazid) initially and the patient used to take it for 6 months od (takes one tablet per day). First, we encourage patients to take the drugs and we assure them that we are not treating TB but it’s a preventive therapy even when the drug may look to be TB treatment drug but its not a full dose so are not treating TB and then give the possible side effects to those drugs. We know that TPT, TB and most HIV drugs are hepatotoxic so most of our side effect and caution is upon liver symptoms. It’s hard to follow up baselines because we don’t know their liver status before they start TPT, so we just alert them incase you see any symptoms come back to the clinic and we change the drugs. In most cases we tell them about the skin rash, yellowing of eyes, abdominal pains and those are the main side effects.

**Interviewer: How do you educate your patients about the effects of hepatotoxicity and the importance of monitoring**

**HW01:** So, like I had said we don’t tell them much about the risks associated because if you tell patient that this drug will damage your liver then they won’t take drugs they will say I rather die to TB than my liver dying. So, In order to protect them from TB so we tell them the drug may affect the liver and actually most of the people who don’t take TPT say “my friend died because of TPT drugs” so it’s quite challenging so we just give them hints and say if you see these symptoms it means the drug is treating your liver badly so come back. So, we don’t tell them about the percentage risk. On the importance of monitoring, we tell them that the drugs treat our body differently and the fact that they are taking ARVs, they have this at the back of their minds. So, we tell them these drugs will prevent from getting TB however they have these side effects and not every person will get these side effects but in case you see for example yellowing of eyes, skin rash, abdominal distention then know that your body is not responding well to the drug however this drug can also some other side effects. We warn them from major signs of liver effect but the rest like yellowing of urine, dryness of throat, and palpitation, we tell you the body is responding to the drug and incase these signs persist for 1-2 weeks come back. But for the monitoring we don’t really tell them we are monitoring so we do it without them knowing so if they come with an issue, we attend to them as we are monitoring without their notice.

**Interviewer: Are there specific barriers you face when conveying this information.**

**HW01:** The main barrier are, One is Pill burden, and the patients will tell you I am already on ARVs and may be hypertensives then again I need they said I don’t feel like taking this drug. And even patients will ask you “if I don’t take this drug will I die of TB” so they tell you “ I don’t need it” so convincing them that they need these drugs is quite challenging. Also, if they don’t have any family history of TB or they have never had someone close to them having TB or they don’t what is going in the world about TB in relation to HIV, it’s quite challenging to convince them. Most of the people who have been at ART, they assume they are continuing the treatment and if you don’t counsel me well, they might not take the drug and when you ask them if they took the drug, they will tell you it home and I took only one drug and also there are those who get the side effect and stop the drugs and don’t see the doctor. Sometimes someone get itching and immediately stops the drugs and now for us we would like to see if the rash is due to TPT or the brand of ARVs they are taking so it’s challenging, but the main problem is pill burden.

**Interviewer: In your opinion what would be the reason for patients not complying with hepatotoxicity monitoring**

**HW01** : Firstly, it depend on how you explain to them if you tell them I will be doing LFTs every two weeks or every month, they will want to know why you are doing these tests and most times if you tell them you are monitoring, patient will want to know why so its hard to explain to non-medical people and patients know that they only need test when they are sick so even if you don’t tell them you are monitoring they will have it at the back of their mind that they liver is dying and that’s why they are doing these tests. Another problem is we don’t do these tests here and the patient has to incur costs so most of them are detached from the tests, so they always say you are giving me a drug and its monitoring needs me to pay for the tests so most of them chill the tests and that’s the main problem.

**Interviewer: Is there system based challenges that clinicians encounter when implementing hepatotoxicity monitoring?**

**HW01** : Here at ISS if you want to do the tests and like those times when we had reagents, you used to order the tests and they do them immediately. Now that we don’t have the stock in our reach the clients have to pay out of pocket and that’s challenging however bad the symptoms are if they don’t have the money then they will tell you I don’t have the money. So, it’s a challenge to the clinicians because even when you see the patient has signs of liver injury and you request for tests, they will tell you we don’t have the money and you put the patient on pressure to do the tests and even if they don’t respond you have nothing to do. Here at ISS, the patients are monitored via phone calls, and we don’t do the tests routinely and when you enrol the patients on TPT and is asymptomatic you will keep calling the patient to ask them of any side effects so we monitor via phone calls or when they come for a visit. But if we are enrolling a patient with symptoms, we do the baseline tests and for those asymptomatic its hard to do the tests before enrolling them and then you tell them to pay again when they get side effects.

How do you think your systems can be improved to enhance hepatotoxicity monitoring.

Accessibility of reagents to the facility so that the tests are routine and if you are doing for the monitoring like for the third time, we already have baseline tests in your files. You find even a cbc for 15000, patients can not afford now imagine LFTs and you doing it every month. So, If the facilities can have access to LFTs and the reagent, that would improve the monitoring process. Right now, we educate them about the symptoms because those can be easily seen but ideally, they are not sufficient without the tests. So of course, they will come with symptoms and even ask what this is if there liver is okay because you have not done the tests.

**Interviewer: Anything you would like to share with me about TPT and hepatotoxicity monitoring**.

**HW01** : Well, throughout the experience I have with patients taking TPT, they are side effects that are not designated to TPT, and if you ask someone, there are signs cited yet people get them when they take TPT although we have different body responses and pharmacodynamics but I fell like the people manufacturing TPT need to explore more about its effects not only on the liver but also other organs. The truth is when you take them off TPT, those symptoms go away and patients are always presented with weird symptoms that are not documented for TPT, so I feel there is still a lot to learn about TPT. For hepatotoxicity monitoring, since we know the drugs are hepatotoxic, all patients should have baseline tests so that if someone is presented with liver damage, then we can know it’s caused by TPT. Most patients are mostly taking many drugs so Ideally, we need to know the cause incase of liver damage and sometimes patients may appear asymptomatic, yet they have a liver damage. So ideally, we need to know the liver status before enrolling these patients for TPT.
