# Supplementary material for: Prevalence, perceptions and factors associated with non-adherence to hepatotoxicity monitoring among people living with HIV on tuberculosis preventive treatment at Mulago ISS clinic
Source: PLoS One. 2026 Mar 30;21(3):e0345662. doi: 10.1371/journal.pone.0345662 (PMC13035147; doi:10.1371/journal.pone.0345662)
Supplement: S4 File — (PDF) [file pone.0345662.s004.pdf]

PATIENT INTERVIEW GUIDE-ENGLISH VERSION.

Greetings.

My name is Kevin Naturinda a principal investigator of the study. On behalf of the research team, we are very grateful for your acceptance to participate in the interview. The purpose of the study is to determine the prevalence, perception and factors associated with non-compliance with hepatotoxicity monitoring and among adult HIV patients on ART and TPT attending Mulago ISS clinic. The information that you will provide, will enable us explore the reasons of non-compliance to hepatotoxicity monitoring and thus stress out challenges that can be rectified by the concerned authorities. Kindly give me your attention for not more than an hour and respond to the questions that will be asked.

Note:

- Your participation in the study is entirely voluntary and you have a right to withdrawal at any point you feel like without any penalty.
- Kindly note that the interview will be recorded for future reference to enable accurate capturing of the information that will be discussed in the interview. The audio records will only be accessed by authorized members of the research team who will respect your privacy and confidentiality.
- Kindly feel free to raise your questions or concerns during the interview.

Interviewee introduction and assignment of a unique identifier.

Guiding questions;

1. What do you understand by hepatotoxicity and TB preventive Therapy?

2. What information were given regarding the potential side effects when you started TPT?
3. Did you feel like you were adequately explained to by your clinician about TPT and its side effects?
4. What are your thoughts on the need to monitor for hepatotoxicity?
5. What could be the barriers that made it difficult for you to comply with hepatotoxicity monitoring recommendation?
6. In your opinion, what do you think could be done to improve on compliance with monitoring need?
7. Is there anything you would wish to share with me about TPT, hepatotoxicity monitoring or any other suggestion?

CLINICIAN INTERVIEW GUIDE

Greetings.

My name is Kevin Naturinda a principal investigator of the study. On behalf of the research team, we are very grateful for your acceptance to participate in the interview. The purpose of the study is to determine the prevalence, perception and factors associated with non-compliance with hepatotoxicity monitoring and among adult HIV patients on ART and TPT attending Mulago ISS clinic. The information that you will provide, will enable us explore the reasons of non-compliance to hepatotoxicity monitoring and thus stress out challenges that can be rectified by the concerned authorities. Kindly give me your attention for not more than an hour and respond to the questions that will be asked.

Note:

- Your participation in the study is entirely voluntary and you have a right to withdrawal at any point you feel like without any penalty.
- Kindly note that the interview will be recorded for future reference to enable accurate capturing of the information that will be discussed in the interview. The audio records will only be accessed by authorized members of the research team who will respect your privacy and confidentiality.
- Kindly feel free to raise your questions or concerns during the interview.

Interviewee introduction and assignment of a unique identifier.

Guiding questions;

1. What are some of the key points do you emphasize to your patients before you enroll them on TPT?

2. In your discussion, how do you educate your patients about TPT, potential risk of hepatotoxicity and the importance of monitoring?
3. Are there specific barriers that you face in conveying this message to your patients?
4. In your opinion, what could be the reasons for patients not complying with hepatotoxicity monitoring?
5. Are there system level limitations that clinicians encounter in implementing hepatotoxicity monitoring for patients on TPT?
6. How can healthcare systems be improved to enhance compliance to hepatotoxicity monitoring?
7. Is there anything you would wish to share with me about TPT, hepatotoxicity monitoring or any other suggestion?

Thanks for your participation.

LABORATORY PERSONNEL INTERVIW GUIDE

Greetings.

My name is Kevin Naturinda a principal investigator of the study. On behalf of the research team, we are very grateful for your acceptance to participate in the interview. The purpose of the study is to determine the prevalence, perception and factors associated with non-compliance with hepatotoxicity monitoring and among adult HIV patients on ART and TPT attending Mulago ISS clinic. The information that you will provide, will enable us explore the reasons of non-compliance to hepatotoxicity monitoring and thus stress out challenges that can be rectified by the concerned authorities. Kindly give me your attention for not more than an hour and respond to the questions that will be asked.

Note:

- Your participation in the study is entirely voluntary and you have a right to withdrawal at any point you feel like without any penalty.
- Kindly note that the interview will be recorded for future reference to enable accurate capturing of the information that will be discussed in the interview. The audio records will only be accessed by authorized members of the research team who will respect your privacy and confidentiality.
- Kindly feel free to raise your questions or concerns during the interview.

Interviewee introduction and assignment of a unique identifier.

Guiding questions;

1. How understand by term hepatotoxicity monitoring for patients among patients on TPT?
2. How often do you receive patients requested to do this laboratory test?

3. Are there any trends or patterns you have observed in non-compliance with sample submission?
4. What challenges, if any do you encounter in ensuring accurate and timely monitoring?
5. Are there any suggestions for improvement in the system in order to serve your patients better?
6. How do you communicate results and relevant information to other healthcare workers?
7. In your opinion, how can collaborations between laboratory personnel, clinicians and patients be improved to ensure compliance with hepatotoxicity monitoring?

Thanks for your participation

LUGANDA VERSION OF PATIENT’S INTERVIEW GUIDE

Mulamusa.

Amannya gange nze Kevin Naturinda omunoonyereza omukulu mu kunoonyereza kuno. Ku lwa ttiimu y’abanoonyereza, tusiima nnyo olw’okukkiriza okwetaba mu mboozi eno.

Ekigendererwa ky’okunoonyereza kuno kwe kuzuula obungi, endowooza n’ensonga ezikwatagana n’obutagoberera kulondoola butwa mu kibumba ne mu balwadde ba siriimu abakulu abali ku ART ne TPT abagenda mu ddwaaliro lya Mulago ISS.

Amawulire g’ogenda okuwa, gajja kutusobozesa okunoonyereza ku nsonga eziviirako obutagoberera kulondoola butwa mu kibumba era bwe tutyo ne tussa essira ku kusoomoozebwa okuyinza okutereezebwa ab’obuyinza abakwatibwako.

Nsaba okumpuliliza okumala essaawa ezitassukka emu era muddemu ebibuuzo ebigenda okubuuzibwa.

Note:

•Okwetaba kwo mu kunoonyereza kwa kyeyagalire kwonna era olina eddembe okuvaamu mu kiseera kyonna ky’owulira nga tolina kibonerezo kyonna.

•Mutegeere nti yintaviyu ejja kukwatibwa okusobola okukozesebwa mu biseera eby’omu maaso okusobozesa okukwata obulungi amawulire agagenda okuteesebwako mu yintaviyu. Ebiwandiiko by’amaloboozi bijja kufunibwa abantu bokka abakkirizibwa mu ttiimu y’abanoonyereza abajja okussa ekitiibwa mu by’ekyama byo n’ebyama byo.

•Mu ngeri ey’ekisa wulira nga oli waddembe okuleeta ebibuuzo byo oba ebikweraliikiriza mu kiseera ky’okubuuza ebibuuzo.

Okwanjula n‟okugaba ekintu eky‟enjawulo ekimanyisa abalwadde.

Ekitabo ky‟abalwadde;

1.Kiki ky‟otegeera ku butwa bw‟ekibumba n‟obujjanjabi obuziyiza TB?

2.Mawulire ki agaaweebwa ku bikwata ku biyinza okuvaamu nga otandise TPT?

3.Wawulira ng’omusawo wo yakunyonyola bulungi ku TPT n’ebizibu ebivaamu?

4.Olowooza ki ku bwetaavu bw’okulondoola obutwa mu kibumba?

5.Biki ebiyinza okuba ebiziyiza ebyakukaluubiriza okugoberera ekiteeso ky‟okulondoola obutwa mu

kibumba?

6.Mu ndowooza yo, olowooza kiki ekiyinza okukolebwa okulongoosa ku kugoberera obwetaavu bw‟okulondoola?

7.Waliwo ky'oyagala okugabana nange ku TPT, okulondoola obutwa mu kibumba oba okuteesa okulala kwonna?

Mwebale nnyo okwetabamu
