# Supplementary material for: Prevalence, perceptions and factors associated with non-adherence to hepatotoxicity monitoring among people living with HIV on tuberculosis preventive treatment at Mulago ISS clinic
Source: PLoS One. 2026 Mar 30;21(3):e0345662. doi: 10.1371/journal.pone.0345662 (PMC13035147; doi:10.1371/journal.pone.0345662)
Supplement: S5 File — (PDF) [file pone.0345662.s005.pdf]

EXTRACTION TOOL

Name of ART Clinic …………………………………….	Record number ……………………………….					
Date of extraction	…………………………………………					
Name of research assistant	…………………………………………					
Patient identification number						
1. Gender (tick where appropriate)	Male ☐		Female ☐			
2. Age (years)						
3. Occupation						
4. Residence						
5. Date of TPT enrollment						
6. Level of income						
7. Level of education (tick where appropriate)	Primary ☐		Secondary ☐	Tertiary ☐	No education ☐	
8. History of alcohol use	Yes ☐		No ☐			
9. Baseline count	viral load…………..		CD4……………………..			
10. History of co-morbidities	Liver disease ☐		Hypertension ☐	Diabetes ☐	None ☐	
11. Hepatitis B status	Positive ☐		Negative ☐			
12. TPT regimen						
13. ART regimen						
14. Request of LFTs	Yes ☐		No ☐			
15. LFTs performed	Baseline	Yes ☐ No ☐	3 months	Yes ☐ No ☐	Other time	Yes ☐ No ☐
END						
